# Safety and Feasibility of MitraClip Implantation in Patients with Acute Mitral Regurgitation after Recent Myocardial Infarction and Severe Left Ventricle Dysfunction

**DOI:** 10.3390/jcm10091819

**Published:** 2021-04-22

**Authors:** Dan Haberman, Rodrigo Estévez-Loureiro, Tomas Benito-Gonzalez, Paolo Denti, Dabit Arzamendi, Marianna Adamo, Xavier Freixa, Luis Nombela-Franco, Pedro Villablanca, Lian Krivoshei, Neil Fam, Konstantinos Spargias, Andrew Czarnecki, Isaac Pascual, Fabien Praz, Doron Sudarsky, Arthur Kerner, Vlasis Ninios, Marco Gennari, Ronen Beeri, Leor Perl, Haim Danenberg, Lion Poles, Sara Shimoni, Sorel Goland, Berenice Caneiro-Queija, Salvatore Scianna, Igal Moaraf, Davide Schiavi, Claudia Scardino, Noé Corpataux, Julio Echarte-Morales, Michael Chrissoheris, Estefanía Fernández-Peregrina, Mattia Di Pasquale, Ander Regueiro, Carlos Vergara-Uzcategui, Andres Iñiguez-Romo, Felipe Fernández-Vázquez, Danny Dvir, Maurizio Taramasso, Mony Shuvy

**Affiliations:** 1 Heart Center, Kaplan Medical Center, Affiliated to the Hebrew University, Jerusalem 9190501, Israel; Lion_P@clalit.org.il (L.P.); sarah_s2@clalit.org.il (S.S.); Sorel_G@clalit.org.il (S.G.); 2 Interventional Cardiology Unit, Hospital Álvaro Cunqueiro, 36321 Vigo, Spain; roiestevez@hotmail.com (R.E.-L.); bcanque@gmail.com (B.C.-Q.); andres.iniguez.romo@sergas.es (A.I.-R.); 3 Interventional Cardiology Unit, Complejo Asistencial Universitario de Leon, 24071 Leon, Spain; tomasbenito@outlook.com (T.B.-G.); juliocecharte@gmail.com (J.E.-M.); ffernandez@secardiologia.es (F.F.-V.); 4 Cardiovascular Surgery Department, San Raffaele University Hospital, 20132 Milan, Italy; denti.paolo@hsr.it (P.D.); lanci82@gmail.com (D.S.); 5 Interventional Cardiology Unit, Hospital Sant Pau i Santa Creu, 08041 Barcelona, Spain; dabitarza@gmail.com (D.A.); efernandezperegrina@gmail.com (E.F.-P.); 6 Cardiac Catheterization Laboratory, ASST Spedali Civili di Brescia, 25123 Brescia, Italy; mariannaadamo@hotmail.com (M.A.); m.dipa91@gmail.com (M.D.P.); 7 Interventional Cardiology Unit, Hospital Clinic, 08036 Barcelona, Spain; xavierfreixa@hotmail.com (X.F.); anderregueiro@gmail.com (A.R.); 8 Hospital Clínico San Carlos, Instituto de Investigacion Sanitaria San Carlos, IdISSC, 28040 Madrid, Spain; luisnombela@yahoo.com (L.N.-F.); carting1@gmail.com (C.V.-U.); 9 Interventional Cardiology, The Center for Structural Heart Disease, Henry Ford Hospital, Detroit, MI 48202, USA; pedrovillablanca@hotmail.com; 10 Department of Cardiology, Kantonsspital Baden, 5404 Baden, Switzerland; liank@me.com (L.K.); Igalmoaraf@gmail.com (I.M.); 11 Division of Cardiology, St. Michael’s Hospital, University of Toronto, Toronto, ON M5B 1W8, Canada; neil.fam@unityhealth.to; 12 Department of Transcatheter Heart Valves, HYGEIA Hospital, 15123 Athens, Greece; kspargias@hygeia.gr (K.S.); mchrissoheris@hotmail.com (M.C.); 13 Schulich Heart Centre, Sunnybrook Health Sciences Centre, University of Toronto, Toronto, ON M4N 3M5, Canada; Andrew.Czarnecki@sunnybrook.ca; 14 Department of Cardiology, Hospital Universitario Central de Asturias, 33011 Oviedo, Spain; ipascua@live.com; 15 Inselspital, Bern University Hospital, University of Bern, 3010 Bern, Switzerland; Fabien.Praz@insel.ch (F.P.); noe.corpataux@insel.ch (N.C.); 16 Cardiovascular Institute, Baruch Padeh Medical Center, Poriya 1520800, Israel; DSudarsky@poria.health.gov.il; 17 Department of Cardiology, Rambam Medical Center, and B. Rappaport Faculty of Medicine, Technion Medical School, Haifa 3109601, Israel; a_kerner@rambam.health.gov.il; 18 Department of Cardiology, Interbalkan European Medical Center, 55535 Thessaloniki, Greece; vninios@gmail.com; 19 Department of Cardiovascular Surgery, IRCCS Centro Cardiologico Monzino, 20138 Milan, Italy; Marco.Gennari@usz.ch; 20 Heart Valve Clinic, University Hospital of Zurich, 8006 Zurich, Switzerland; Salvatore.Scianna@usz.ch (S.S.); Maurizio.Taramasso@usz.ch (M.T.); 21 Heart Institute, Hadassah-Hebrew University Medical Center, Jerusalem 91120, Israel; ronenbe@ekmd.huji.ac.il (R.B.); danen040@gmail.com (H.D.); monysh@gmail.com (M.S.); 22 Cardiology Department, Rabin Medical Center and the “Sackler” Faculty of Medicine, Tel-Aviv University, Tel-Aviv 49100, Israel; leorperl@gmail.com; 23 Department of Cardiology, Joan XXIII University Hospital, 43005 Tarragona, Spain; claudia.scardino85@gmail.com; 24 Jesselson Integrated Heart Centre, Shaare Zedek Medical Center, Hebrew University, Jerusalem 9103102, Israel; danny.dvir@gmail.com

**Keywords:** mitral regurgitation, percutaneous mitral valve repair, acute myocardial infarction, left ventricle dysfunction

## Abstract

Patients with severe mitral regurgitation (MR) after myocardial infarction (MI) have an increased risk of mortality. Transcatheter mitral valve repair may therefore be a suitable therapy. However, data on clinical outcomes of patients in an acute setting are scarce, especially those with reduced left ventricle (LV) dysfunction. We conducted a multinational, collaborative data analysis from 21 centers for patients who were, within 90 days of acute MI, treated with MitraClip due to severe MR. The cohort was divided according to median left ventricle ejection fraction (LVEF)—35%. Included in the study were 105 patients. The mean age was 71 ± 10 years. Patients in the LVEF < 35% group were younger but with comparable Euroscore II, multivessel coronary artery disease, prior MI and coronary artery bypass graft surgery. Procedure time was comparable and acute success rate was high in both groups (94% vs. 90%, *p* = 0.728). MR grade was significantly reduced in both groups along with an immediate reduction in left atrial V-wave, pulmonary artery pressure and improvement in New York Heart Association (NYHA) class. In-hospital and 1-year mortality rates were not significantly different between the two groups (11% vs. 7%, *p* = 0.51 and 19% vs. 12%, *p* = 0.49) and neither was the 3-month re-hospitalization rate. In conclusion, MitraClip intervention in patients with acute severe functional mitral regurgitation (FMR) due to a recent MI in an acute setting is safe and feasible. Even patients with severe LV dysfunction may benefit from transcatheter mitral valve intervention and should not be excluded.

## 1. Introduction

Mitral regurgitation (MR) complicating acute Myocaridial Infarction (MI) can be the result of papillary muscle rupture (Primary) or imbalance between closing and tethering forces (Functional). Primary MR in this acute setting is a medical emergency often requires emergent intervention [1]. Functional mitral regurgitation (FMR) usually develops over the course of days and is associated with adverse outcomes [2]. Currently, medical therapy is the mainstay approach for this condition. However, some patients remain very symptomatic with prolonged hospitalization and frequent heart failure (HF) admissions [3].

Data obtained from large-scale registries suggest that percutaneous transcatheter edge-to-edge mitral valve repair implantation with MitraClip device improves functional capacity and quality of life [4,5,6,7]. Furthermore, the recent randomized trial entitled, ‘Cardiovascular Outcomes Assessment of the MitraClip Percutaneous Therapy for Heart Failure Patients with Functional Mitral Regurgitation’ (COAPT) showed that MitraClip improved clinical outcomes, including mortality in patients with severe FMR, when compared to medical therapy alone [8]. Conversely, the results of the ‘Multicentre Study of Percutaneous Mitral Valve Repair MitraClip Device in Patients with Severe Secondary Mitral Regurgitation’ (MITRA-FR) illustrated that enrolled patients with lower LVEF showed no clinical benefit from the MitraClip procedure in an advanced HF population [9], thereby suggesting that patients with lower LVEF may benefit less from the MitraClip procedure.

Focusing on patients with reduced left ventricle (LV) function, previous studies have addressed this issue with mixed results. In the German transcatheter mitral valve interventions (TRAMI) registry, the clinical benefit for patients with severely reduced LV function was comparable to patients with preserved LV [6]. However, a recent paper by Pascual et al. showed that patients in the LVEF > 30% group had better clinical outcomes [10]. Percutaneous edge-to-edge repair for functional MR was recently implemented in the American Heart Association 2020 clinical guidelines [11]. However, it is still not clear how effective it is for patients with a severe reduction in LV function. While the positive value of MitraClip implantation on outcomes in chronic severe symptomatic ischemic FMR was shown before [12], data regarding patients with severe acute ischemic FMR, who are generally not included in registries or in randomized trials, are lacking, especially in patients with severely reduced LV function.

Our group previously published two case series showing that the MitraClip procedure is safe in patients with pulmonary congestion due to severe functional MR complicating MI [13,14]. MR reduction was significant with favorable hemodynamics and clinical improvement. The mortality rate after 30 days was as low as 10%. A recent publication by our group showed that even patients in cardiogenic shock may benefit from percutaneous edge-to-edge repair [15].

However, it is unknown how effective the MitraClip procedure is in severe LV dysfunction patients in an acute setting.

## 2. Methods

The International Registry of MitraClip in Acute Mitral Regurgitation following acute myocardial infarction (IREMMI) was established to assess the safety and outcomes of patients who underwent MitraClip in an acute setting following MI. In this multinational, multicenter retrospective registry, we evaluated the short and intermediate outcomes based on left ventricle ejection fraction. The procedure was performed in 21 centers in Europe, North America and Israel using the MitraClip device (Abbott, Menlo Park, CA, USA).

### 2.1. Study Population

We included patients who were treated with the MitraClip device due to at least moderate to severe (3+) MR, within 90 days of acute myocardial infarction (MI) between January 2014 and January 2020. Although treated medically, patients remained symptomatic (NYHA class > 3) and were evaluated by a multidisciplinary team of a non-interventional cardiologist, an interventional cardiologist and a cardiothoracic surgeon. All patients were deemed to be high risk for surgery and therefore a MitraClip procedure was performed in order to allow recovery. Patients who were anatomically unsuitable for edge-to-edge mitral valve repair were excluded. We evaluated immediate, 3-month and 1-year outcomes in those patients

### 2.2. Echocardiographic Evaluation

The severity of MR, left ventricle ejection fraction (LVEF), pulmonary artery pressure (PAP) and Mitral valve (MV) gradient were measured and graded according to the American Society of Echocardiography guidelines [16,17,18]. The severity of MR was assessed with an integrated multiparametric visual evaluation tool in accordance with standard clinical practice (incorporating 2D, spectral and color Doppler images), using an ordinal scale (grading 0− no MR, 1+ mild MR, 2+ moderate MR, 3+ moderate to severe MR, 4+ severe MR). A transesophageal echocardiogram (TEE) was completed in all patients prior to the procedure. MR grade, Mitral valve area (MVA) and MV gradient, calcium at grasping area and coaptation features were assessed in order to evaluate MitraClip feasibility.

### 2.3. MitraClip

A MitraClip procedure was performed under general anesthesia; fluoroscopy and transesophageal echocardiography (TEE) were routinely used for guidance. After transseptal puncture, the delivery system was advanced to the left atrium (LA), LV and retracted back to grasp the anterior and posterior leaflets. Procedural success was defined as the successful grasping of the anterior and posterior leaflets together with a reduction of at least two MR grades. V-wave and left atrial pressures (LAP) were measured and recorded immediately before and after MitraClip implantation.

### 2.4. Outcomes

Procedural and clinical adverse events during follow-up were defined according to the Mitral Valve Academic Research Consortium (MVARC) [13]. Outcomes of interest were procedural success, safety, hospital discharge, clinical outcomes and hemodynamics. Acute procedural success was defined as successful implantation of one or more clips with a reduction of the MR to <2+. Safety outcomes included procedural and periprocedural complications, namely, clip detachment, cerebrovascular event, MI, bleeding requiring transfusion and cardiac tamponade and urgent cardiovascular surgery. Clinical outcomes included weaning from mechanical ventilation and/or an LV support device and an NYHA class at follow-up. Hemodynamic parameters, LAP and V-wave were measured during the procedure. In cases where right heart catheterization was used, pulmonary artery wedge pressure (PCWP) was measured simultaneously with the procedure. After discharge, patients were followed up by individual centers in outpatient clinic visits in which clinical evaluation and echocardiography were performed.

### 2.5. Statistical Analysis

Our analyses were performed using the entire cohort, drawing comparisons between patients with EF lower or higher than 35%. Patient characteristics are reported according to variable properties.

Categorical variables (ex. MR grade) are reported as % (*n*), and differences between subgroups were tested, when appropriate, using the chi-square test of Fischer’s exact test. Continuous variables (ex. LVEF, PCWP, V-wave, sPAP) are reported according to their distribution. Those with a normal distribution are reported as mean (±standard deviation), and differences between subgroups were tested using the student’s T-test. Those without a normal distribution are reported as median (interquartile range), and differences between subgroups were tested using the Mann-Whitney U. A p-value less than 0.05 was considered to be statistically significant. Kaplan-Meier estimates were used to calculate survival curves, which were adjusted to age and compared using the log-rank test. All clinical events were analyzed by time-to first event for Kaplan-Meier analysis. The IBM Statistical Package for the Social Sciences (SPSS) Statistics 26.0 (IBM Corp., Armonk, NY, USA) was utilized to perform the analyses.

## 3. Results

From January 2014 to January 2020, 105 patients were included in the registry.

### 3.1. Patient Characteristics

The mean age of patients was 70.5 ± 10.3 years and 50% were female. All patients had at least MR grade 3 before the intervention. Patients suffered pulmonary congestion despite initial medical therapy, including intravenous diuretics. Of the patients, 52 (55%) were in cardiogenic shock and were further supported by an intra-aortic balloon pump (IABP) or intravenous vasopressors, whilst 41 patients (43%) required mechanical ventilation unrelated to the procedure. The patient population was divided into two groups based on the median LVEF of 35%. The LVEF < 35% group included 47 patients, whilst LVEF ≥ 35% included 58 patients with a mean LVEF of 26 ± 6% and 44 ± 9%, *p* < 0.01, respectively.

Baseline characteristics are listed in Table 1. Patients in the LVEF < 35% group were younger when compared to patients with LVEF ≥ 35% (68.4 ± 9.1 vs. 72.4 ± 11.0, *p* = 0.05), as well as having a higher body–mass index (BMI) (27.9 ± 4.7 vs. 25.5 ± 5.3, *p* = 0.04). There was no difference in other risk factors for cardiovascular disease, past MI, past Coronary Artery Bypass Graft (CABG) or in the surgical risk assessment by Euroscore II.

Out of 105 patients, 75 presented with STEMI but there was no significant difference between the two groups (66% vs. 76%, respectively, *p* = 0.14). In both groups there was a high rate of multi-vessel disease (80% vs. 78%, *p* = 0.63). The common infarct related artery (IRA) was the LAD in the LVEF < 35% group (53% vs. 22%), and the RCA in the LVEF ≥ 35% group (41% vs. 11%, *p* < 0.01).

The anterior LV wall was involved in 51% of patients in the LVEF < 35% group vs. 19% of patients in the LVEF ≥ 35% group. Accordingly, the inferior wall was more involved in the LVEF ≥ 35% group (59% vs. 30%, *p* < 0.01).

### 3.2. Procedural and Safety Outcomes

Procedure characteristics, safety and outcomes are listed in Table 2.

**Figure 1:**
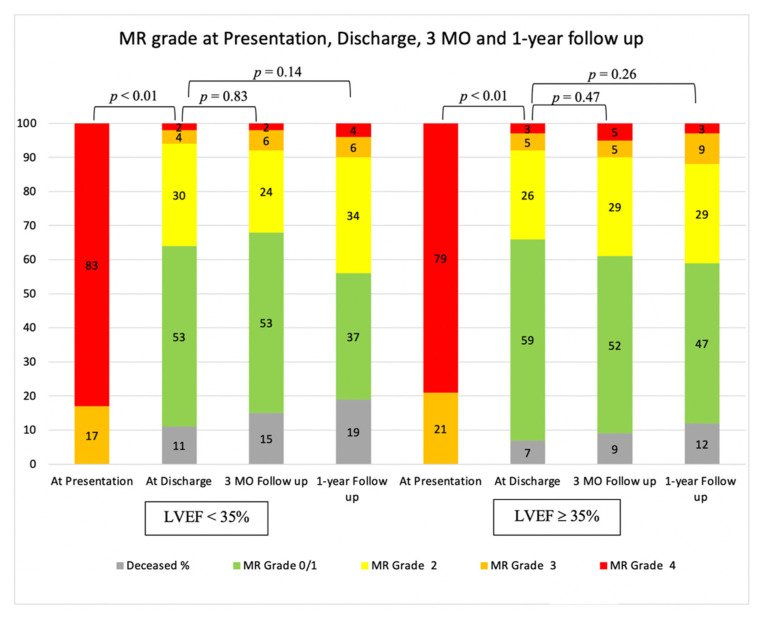
MR grades at presentation, discharge, 3-month and 1-year follow-up. MR was significantly reduced from presentation to discharge in both LVEF groups. MR reduction remains consistent after discharge and throughout follow-up in both groups.

A MitraClip procedure was performed, on average, 27 ± 22 days after the date of the index MI. When drawing comparisons based on LVEF, procedures were delayed in the LVEF ≥ 35% group, where the MitraClip procedure was performed, on average, at 37 ± 26 days vs. 21 ± 18 days, *p* = 0.001 in the LVEF < 35% group. Procedure success was 91.4% (96 out of 105 patients). There was no significant difference between the two LVEF groups, 96% vs. 90%, *p* = 0.73. The procedure’s mean duration was 115 min ± 90 min. There was no difference in duration between the LVEF < 35% group (122 min ± 84 min) and the LVEF ≥ 35% group (109 min ± 95 min), *p* = 0.51. MR reduction to grade 1 was achieved in 64 patients and to 2+ in 32 patients (Figure 1). The grade of MR significantly decreased after the procedure, *p* < 0.01. No difference was observed between the two LV groups. One to four clips were implanted in each case; 42 patients were implanted with a single clip, 52 were implanted with two clips, 9 were implanted with three clips and one patient was implanted with four clips. The mean mitral valve gradient was 3.7 mmHg ± 1.7 and there was no significant difference based on LVEF.

**Figure 2:**
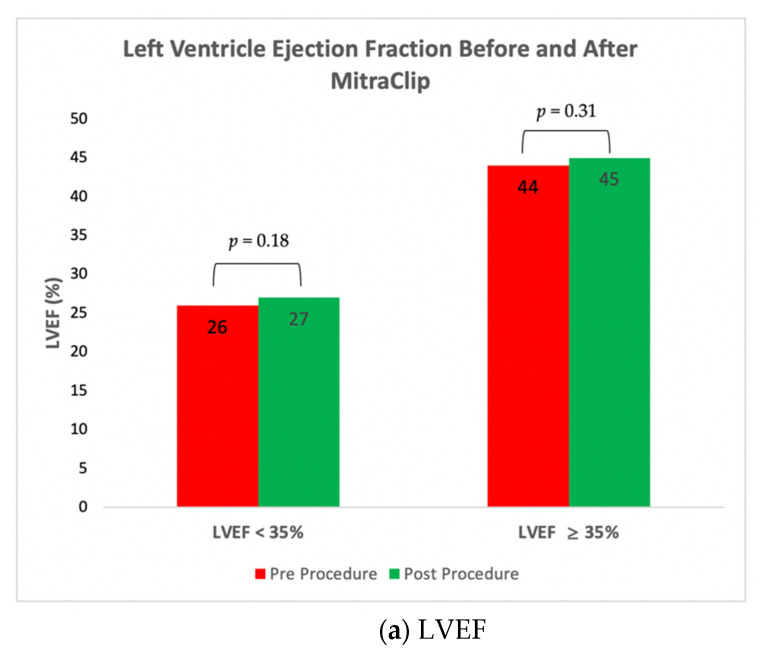

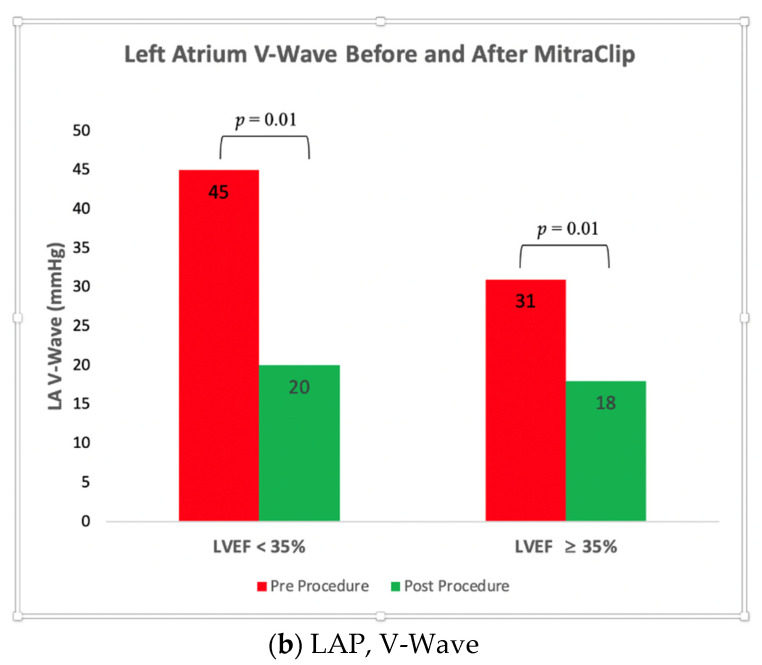
Left Ventricle Ejection Fraction (LVEF) and Left Atrial Pressure (LAP) before and after the procedure. (**a**) There was no significant change in Left ventricle ejection fraction (LVEF) before and after MitraClip procedure in both LVEF < 35% and LVEF ≥ 35% groups; (**b**) There was a significant reduction in LAP before and after MitraClip procedure in both LVEF < 35% and LVEF ≥ 35% groups.

**Figure 3:**
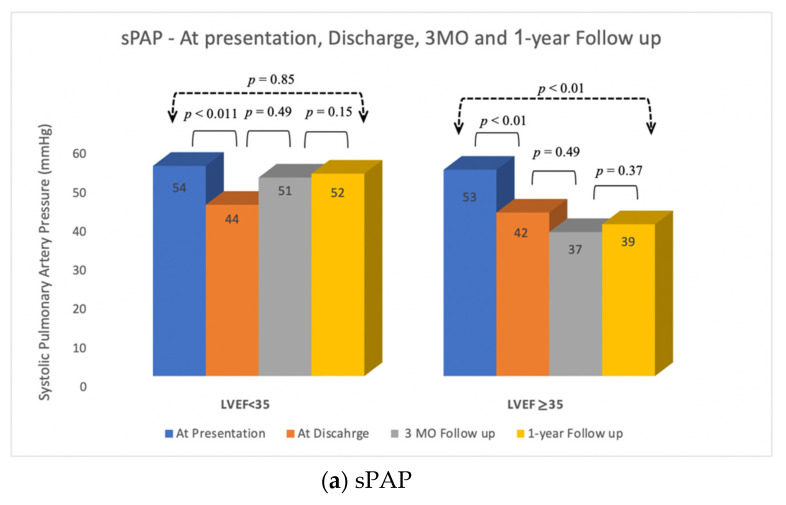

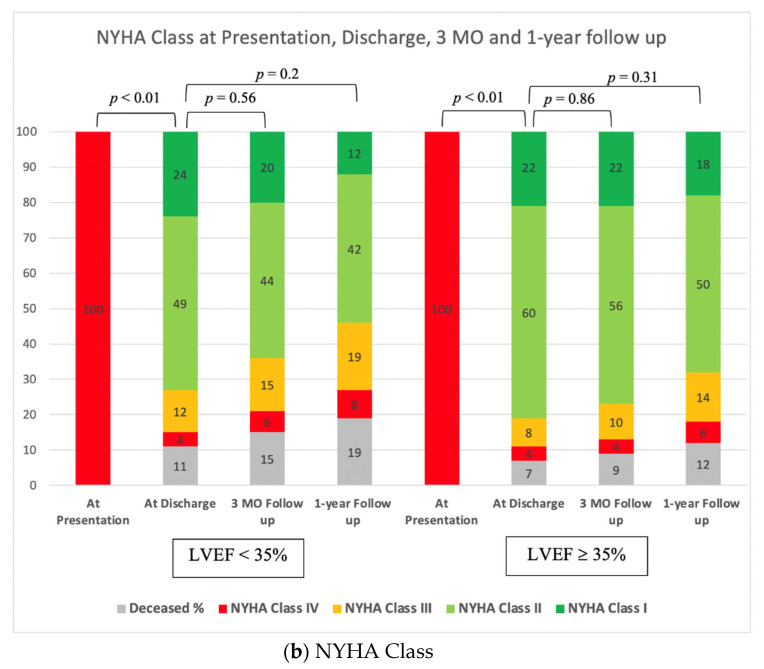
Clinical and Echocardiographic evaluation at presentation, discharge, 3-month and 1-year follow-up. (**a**) Systolic Pulmonary Artery Pressure (sPAP) was significantly reduced from presentation to discharge in both LVEF groups. sPAP reduction from presentation to 1-year follow up was consistent only for patients in the LVEF > 35%. (**b)** NYHA class was significantly improved from presentation to discharge in both LVEF groups. NYHA class improvement remain consistent throughout follow-up in both groups; 36 out of 42 patients in the LVEF < 35% group and 51 out of 54 patients in the LVEF ≥ 35% group had NYHA functional class I or class II at 3-month follow up.

Hemodynamic features from presentation to discharge were improved in both groups. Left atrial V-Wave was reduced from 45.1 ± 15.9 mmHg to 20.2 ± 7.0 mmHg, *p* < 0.01 in the LVEF < 35% group and from 31.3 ± 17.1 mmHg to 17.1 ± 5.7 mmHg, *p* < 0.01, in the LVEF ≥ 35% group (Figure 2a). There was no significant change in EF from presentation to discharge in either group (26% to 27%, *p* = 0.18 and 44% to 45%, *p* = 0.31, respectively) (Figure 2b). SPAP reduction was observed in both groups from presentation to discharge (54.1 ± 14.8 mmHg to 43.9 ± 16.3 mmHg, *p* < 0.01, in the LEVF < 35% group and from 52.9 ± 22.8 mmHg to 41.7 ± 21.7 mmHg, *p* < 0.01, in the LVEF ≥ 35% group) (Figure 3a). Major complications following the procedure were relatively low in both groups (7% vs. 9%, *p* = 0.70).

### 3.3. Mortality Analysis

**Figure 4:**
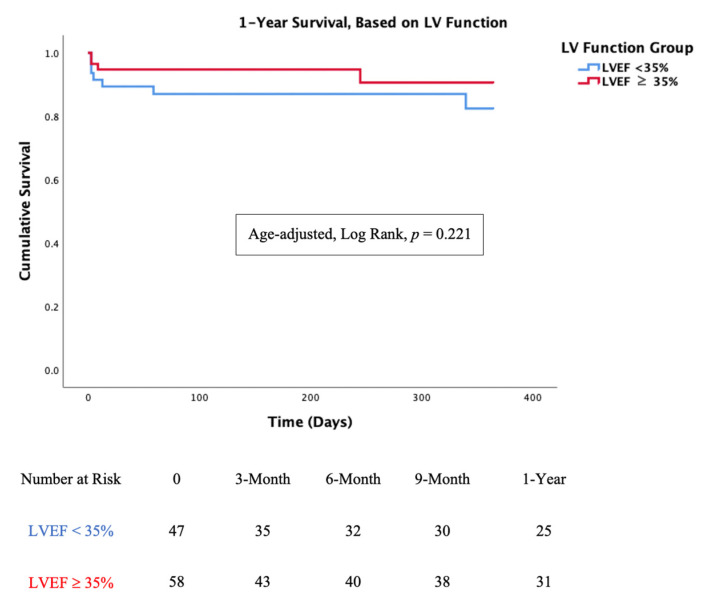
Kaplan-Meier curve for survival, based on LVEF. Left Ventricle Ejection Fraction (LVEF) was not associated with lower or higher mortality rate, Log Rank, *p* = 0.221.

In-hospital mortality was low in both the LVEF < 35% and LVEF ≥ 35% groups (11% vs. 7%, *p* = 0.51). One-year mortality was also low and non-significant when comparing the two groups (19% vs. 12%, *p* = 0.49). Age-adjusted survival curves for mortality are shown in Figure 4. There was no significant difference between the two groups at the 1-year follow-up (Log Rank, *p* = 0.221).

### 3.4. Follow-Up

The median follow-up period was 12 months (IQR 6 to 21).

Follow-up echocardiography after the procedure demonstrated non-significant change in MR from discharge to 3-month and 1-year follow up (Figure 1). This may indicate the durability of the MitraClip procedure, regardless of LV function. Patients benefited from a considerable improvement in functional capacity after the procedure. This improvement was observed throughout the follow-up period (Figure 3b).

As mentioned, significant reduction in sPAP was achieved in both groups after the procedure. During follow-up (3-month and 1-year), there was no significant change in sPAP between the two groups. However, when comparing the pre-procedure sPAP with 1-year sPAP, patients in the LVEF < 35% group did not benefit from sustainable reduction (54 mmHg and 52 mmHg, *p* = 0.85), as opposed to patients in the LVEF ≥ 35% group (53 mmHg to 39 mmHg, *p* < 0.01). This change in hemodynamics between the groups was not translated to a change in functional class at 3-month and 1-year follow-up. Rehospitalization rates at 3-month follow-up were also comparable (19% vs. 16%, *p* = 0.77).

## 4. Discussion

In this international multicenter study, we collected the largest published series of patients who underwent the MitraClip procedure after acute MI complicated by severe MR. We aimed to compare the feasibility of PMVR in patients according to their EF.

There were few significant differences in the basic characteristics between the two EF groups, namely, age and BMI. Almost half of patients required mechanical support and/or were treated with inotropes. In the majority of patients, the salvage MitraClip procedure significantly decreased the MR, as well as the pulmonary hypertension and left atrial V wave; this therefore allowed recovery. Procedural complications were rare, and the clinical condition of most patients was significantly improved as a result of the mitral valve repair. The clinical benefit was maintained through follow up evaluation.

Primary mitral regurgitation in the acute setting is usually due to complete or partial papillary muscle rapture, secondary to ischemia or necrosis, resulting in prolapse or flail leaflet. This condition is accompanied with rapid deterioration and often requires urgent intervention.

Ischemic MR is caused by left ventricular global or regional pathological remodeling due to acute or chronic coronary artery disease [19]. The prevalence of this condition varies between studies and reaches 50% of all MI patients in some series [20]. MR is a central component of the vicious cycle of LV remodeling and HF in MI patients. The regurgitation raises the volume overload and ventricular wall stress, causing further LV dilatation that worsens the MR [19]. Solid data suggests that FMR on presentation, or persistent ischemic FMR, is a negative prognostic factor that is associated with worsened short-term and long-term prognosis [20,21].

Ischemic FMR occurs more frequently with inferior infarction due to geometric distortion in the region of the papillary muscle [22]. Interestingly, in our cohort, 50% of patients presented with anterior MI, suggesting that FMR that is associated with anterior wall infarctions is more likely to require salvage mitral valve intervention. A possible explanation for this is that an anterior wall MI involves more dysfunctional LV and a larger infarct size that causes apical tethering of the valve compared with the localized infarction of the inferior wall. Indeed, MR complicating anterior STEMI was shown to be associated with worse short-term and long-term outcomes than inferior wall infarct [23]. Previous studies have shown that patients at higher risk of developing ischemic FMR are elderly females who have lower EF and multi-vessel CAD [24]. It is reasonable to assume that EF is an important predictor of outcomes after MI and, indeed, data from the Acute Coronary Treatment and Intervention Outcomes Network (ACTION) Registry [25] suggests that EF is directly associated with outcomes among patients after MI: the risk of 1-year mortality was 29.0% in patients with EF ≤ 35%, compared with 13.0% in patients with EF ≥ 55%. On the other hand, Gerber et al. evaluated the prognostic impact of HF after MI in more than 2500 patients and found that mortality was related to HF but did not differ by EF [26].

In the German transcatheter mitral valve interventions (TRAMI) registry, patients were divided into three groups: LVEF < 30%, LVEF 30–50% and LVEF < 50%. It was concluded that the clinical benefit to patients with severely reduced LV function was comparable to patients with preserved LV. In this registry, over 80% of patients in the LVEF < 30% had functional MR^6^. In a recent study published by Pascual et al., patients were divided according to LVEF above and below 30% and showed that patients in the LVEF > 30% group had better clinical outcomes [10].

We found that patients with severe acute MR and LVEF < 35% had similar mortality rates to patients with LVEF ≥ 35%. This finding may suggest that, rather than low EF, severe acute MR is the dominant factor in deterioration and clinical instability and should therefore be treated regardless of EF. However, we cannot exclude that the similar survival rates were related to a lack of statistical power. Revascularization for acute MI can provide improvement of LV function and may decrease the ischemic FMR. Nishino et al. showed that shorter reperfusion time and successful revascularization at presentation were the independent predictors of improvement in FMR [21]. The Acute Myocardial Infarction Contrast Imaging (AMICI) multicenter study showed that a short time to revascularization (<2.5 h) independently predicts reverse remodeling which affects the magnitude of FMR [27].

While the negative prognostic effect of ischemic FMR is well recognized, the impact of surgical repair of MR on LV remodeling and outcomes is still controversial, especially in patients with severely reduced LV function, who are poor candidates for surgery [28,29,30]. Theoretically, the less invasive MitraClip procedure may be considered as an alternative to surgery in high-risk patients. In fact, recent trials suggest that MitraClip therapy improves quality of life and may even reduce mortality among patients with severe chronic ischemic MR [4,6]. However, in most of these clinical trials, patients who had acute or subacute MR due to a recent MI were excluded.

The impact of MitraClip in an acute setting was initially only evaluated in case reports and a small case series [31,32,33,34]. The two largest case series were previously published by our group [13,14]. Our findings confirm that MitraClip is a safe and effective procedure in acute settings for a selected high-risk group, both with LVEF < 35% and LVEF ≥ 35%, and that in most of the patients the effectiveness of mitral intervention was sustainable in long term follow up.

The study has several limitations that merit consideration. First, although several centers were involved, the sample size is relatively small, and the results should be interpreted with caution. Second, our analysis used retrospectively analyzed observational data and therefore the associations between baseline characteristics and outcomes may be confounded by unmeasured variables. Third, in all our cases, MitraClip was performed as a salvage procedure in acutely ill patients. Therefore, the findings cannot be generalized for stable patients who develop ischemic FMR after MI.

Large, multicenter and randomized trials are needed to confirm this data among acute ischemic FMR.

## 5. Conclusions

MitraClip intervention in patients with acute severe FMR due to a recent MI in an acute setting is safe and feasible. Even patients with severe LV dysfunction may benefit from transcatheter mitral valve intervention and should not be excluded.

## Figures and Tables

**Table 1:** Baseline Patient Characteristics.

	Parameter		1	2	
		Total Population	LVEF < 35%	LVEF ≥ 35%	*p*-Value
	*n*	105	47	58	
Demographics					
	Age, years	70.5 ± 10.3	68.4 ± 9.1	72.4 ± 11.0	0.05
	Sex, Female, % (*n*)	50 (53)	43 (20)	57 (33)	0.17
	BMI (Kg/M^2^)	26.5 ± 5.2	27.9 ± 4.7	25.5 ± 5.3	0.04
	Hypertension, % (*n*)	70.5 (74)	68.1 (32)	70.7 (41)	0.83
	Diabetes, % (*n*)	45.7 (48)	51.1 (24)	41.4 (24)	0.33
	Dyslipidemia, % (*n*)	62.9 (66)	70.0 (31)	60.3 (35)	0.69
	COPD, % (*n*)	17.1 (18)	19.1 (9)	15.5 (9)	0.80
	CKD ≥ grade 2, % (*n*)	29.5 (31)	25.5 (12)	32.8 (19)	0.82
	Prior stroke, % (*n*)	13.3 (14)	6.4 (3)	19.0 (11)	0.08
	Prior MI, % (*n*)	55.2 (58)	61.7 (29)	50 (29(	0.24
	Prior CABG, % (*n*)	26.7 (28)	23.4 (11)	29.3 (17)	0.51
	Euroscore 2, %	17.2 ± 16.7	20.1 ± 18.5	14.9 ± 15.0	0.14
Presentation					
	STEMI, % (*n*)	71.4 (75)	66.0 (31)	75.9 (44)	0.25
	Killip Class 3+, % (*n*)	68.6 (72)	78.7 (37)	60.3 (35)	0.06
Echocardiographic					
	MR Grade 4+, % (*n*)	81.0 (85)	83.0 (39)	79.3 (46)	0.80
	Papillary muscle rapture, % (*n*)	5.7 (6)	2.1 (1)	8.6 % (5)	0.22
	Ejection Fraction (%)	35.8 ± 11.9	25.8 ± 5.7	44 ± 8.9	<0.01
	sPAP, mmHg	53.6 ± 18.6	54.4 ± 16.1	53.1 ± 20.3	0.75
Coronary Angiography					
	Multivessel disease, % (*n*)	80 (84)	83.0 (39)	77.6 (45)	0.63
Infarct Related Artery (IRA)	RCA, % (*n*)		11 (5)	41 (24)	<0.01
	LCX, % (*n*)		32 (15)	33 (19)	
	LAD, % (*n*)		53 (25)	22 (13)	
	PCI, % (*n*)	94.3 (99)	95.7 (45)	95.7 (54)	0.69
Wall Involved	Anterior		51 (24)	19 (11)	<0.01
	Inferior		30 (14)	59 (34)	
	Lateral and/or Posterior		19 (9)	22 (13)	
ICCU Status and Treatment					
	Cardiogenic Shock	54.3 (57)	63.8 (30)	46.6 (27)	0.12
	Mechanical Ventilation	42.9 (45)	46.8 (22)	39.7 (23)	0.55
	Vasoactive medication	42.9 (45)	48.9 (23)	37.9 (22)	0.32
	Any MSD	37.1 (39)	42.6 (20)	32.8 (19)	0.32

Abbreviations: BMI—Body Mass Index; CABG—Coronary Artery Bypass Grafting; CAD—Coronary Artery Disease; CKD—Chronic Kidney Disease; COPD—Chronic Obstructive Lung Disease; Euroscore II—European System for Cardiac Operative Risk Evaluation; ICCU—Intensive Coronary Care Unit; LAD—Left Anterior Descending; LCX—Left Circumflex; LVEF—Left Ventricle Ejection Fraction; MSD—Mechanical Support Device; PCI—Percutaneous Coronary Intervention; RCA—Right Coronary Artery; sPAP—Systolic Pulmonary artery pressure; STEMI—ST Elevation myocardial infarction.

**Table 2:** Procedure and Outcomes.

Parameter		1	2	
	Total Population	LVEF < 35%	LVEF ≥ 35%	*p*-Value
*n*	105	47	58	
Procedure				
Procedure Time, min	115 ± 90	122 ± 84	109 ± 95	0.51
Major Complications	6.7 (7)	8.5 (4)	5.2 (3)	0.70
MI to Procedure, days	27 ± 22	35 ± 26	20 ± 17	<0.01
Clips Implanted, mean	1.7 ± 0.7	1.8 ± 0.7	1.6 ± 0.6	0.22
MR > 2 at discharge, % (*n*)	8.6 (9)	8.5 (4)	8.6 (5)	1
MV Gradient post	3.7 ± 1.7	3.7 ± 1.6	3.7 ± 1.8	0.91
Outcomes				
Procedure success	91.4 (96)	93.6 (44)	89.7 (52)	0.73
In hospital mortality	8.6 (9)	10.6 (5)	6.9 (4)	0.51
Major Complications	6.7 (7)	8.5 (4)	5.2 (3)	0.70
Mortality at 3 months	11.4 (12)	14.9 (7)	8.6 (5)	0.50
Rehospitalizations at 3 months	13.3 (14)	19.1 (9)	15.5 (9)	0.77
1-year mortality	15.2 (16)	19.1 (9)	12.1 (7)	0.49

Abbreviations: LVEF—Left Ventricle Ejection Fraction; MI—Myocardial Infarction; MO—Month; MR—Mitral Regurgitation; MV—Mitral Valve.

## Data Availability

The data that supports the findings of this study are available from the corresponding author, D.H., upon reasonable request.
